# Liquid biopsy genotyping in lung cancer: ready for clinical utility?

**DOI:** 10.18632/oncotarget.14613

**Published:** 2017-01-12

**Authors:** Wei-Lun Huang, Yi-Lin Chen, Szu-Chun Yang, Chung-Liang Ho, Fang Wei, David T. Wong, Wu-Chou Su, Chien-Chung Lin

**Affiliations:** ^1^ Department of Internal Medicine, National Cheng Kung University Hospital, College of Medicine, National Cheng Kung University, Tainan, Taiwan; ^2^ Department of Pathology, National Cheng Kung University Hospital, College of Medicine, National Cheng Kung University, Tainan, Taiwan; ^3^ School of Dentistry, University of California, Los Angeles, CA, USA; ^4^ Institute of Clinical Medicine, National Cheng Kung University Hospital, College of Medicine, National Cheng Kung University, Tainan, Taiwan

**Keywords:** ctDNA, CTC, EGFR mutation, T790M

## Abstract

Liquid biopsy is a blood test that detects evidence of cancer cells or tumor DNA in the circulation. Despite complicated collection methods and the requirement for technique-dependent platforms, it has generated substantial interest due, in part, to its potential to detect driver oncogenes such as epidermal growth factor receptor (EGFR) mutants in lung cancer. This technology is advancing rapidly and is being incorporated into numerous EGFR tyrosine kinase inhibitor (EGFR-TKI) development programs. It appears ready for integration into clinical care. Recent studies have demonstrated that biological fluids such as saliva and urine can also be used for detecting EGFR mutant DNA through application other user-friendly techniques. This review focuses on the clinical application of liquid biopsies to lung cancer genotyping, including EGFR and other targets of genotype-directed therapy and compares multiple platforms used for liquid biopsy.

## LIQUID BIOPSY AND TISSUE BIOPSY IN LUNG CANCER

**Figure 1:**
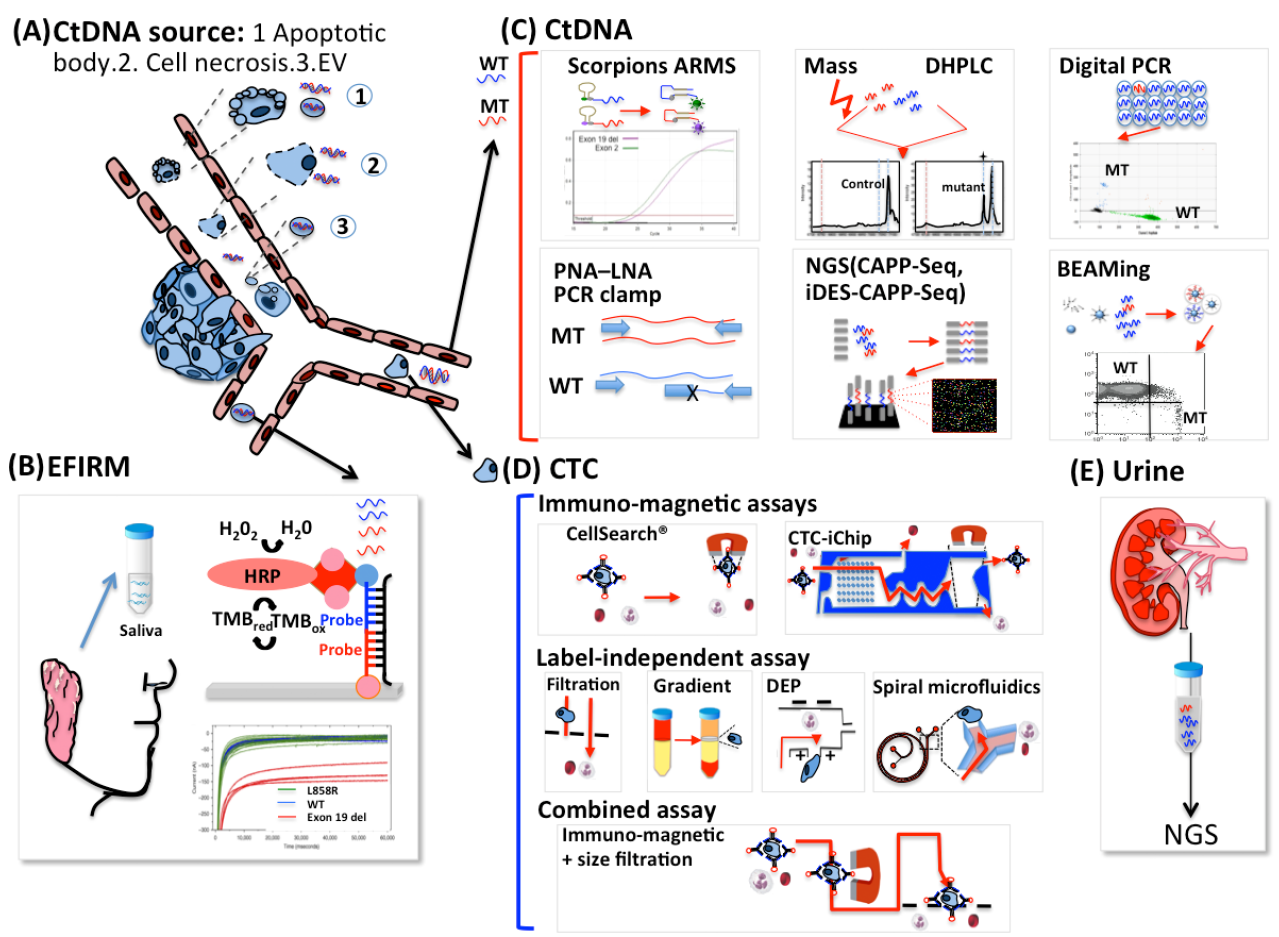
Sources of ctDNA and different platforms for detecting EGFR mutations in patients with lung cancer **A**. Tumor cells constantly release CtDNA into circulation by a variety of mechanisms including cancer cell apoptosis and necrosis because of the rapid cellular turnover in tumors and extracellular vesicles (EVs) actively secreted by living tumor cells. PCR-based platforms are the most commonly used for detecting EGFR mutations, and non-PCR-based platforms such as EFIRM can be used to detect EGFR mutations. **B**. The conjugations between sample DNA, the detector probe, and the capture probe induce a reaction between the HRP-labeled reporter probe and the TMB substrate and generate amperometric signals. **C**. The EGFR genotype can be determined in cDNA by several strategies, including amplifying target alleles (real-time PCR, ARMS/Scorpion assay), suppressing wild-type PCR products (PNA-LNA PCR clamp, peptide nucleic acid-locked nucleic acid PCR clamp), dividing each sample into 20,000 or more discrete subunits before amplification by use of BEAM and digital PCR. DHPLC and MALDI-TOF, under the umbrella of mass spectrometry, analyze DNA mutations after PCR amplification. NGS is uses DNA polymerase catalyzed incorporation of fluorescently labeled nucleotides across millions of fragments in a massively parallel fashion. **D**. Circulating tumor cells can be enriched by label-dependent and label-independent techniques. The label-dependent methods such as magnetic bead (CellSearch) and CTC chips, based on immunomagnetic assays target an antigen using an antibody conjugated to a magnetic bead. Filtration, Ficoll gradient, dielectrophoresis, and spiral microfluidics are based on the physical properties of tumor cells including size, density, electrical properties, and inertial-Dean drag force combinations, respectively, compared with erythrocytes and leukocytes. Combination methods including magnetic bead and filtration can be used to isolate CTCs more efficiently. **E**. Urine DNA derived from circulation was suggested to be mostly low-MW type. EGFR status was analyzed using a PCR method that amplifies short-target DNA fragments using kinetically favorable binding conditions for a wild-type blocking oligonucleotide, followed by massively parallel NGS. EFIRM: Electric field-induced release and measurement. HRP: Horseradish peroxidase; TMB: 3,3’,5,5’-Tetramethylbenzidine; MT: Mutation; WT: wild type; ARMS: Amplification-refractory mutation system/Scorpion assay; PNA-LNA PCR clamp: peptide nucleic acid-locked nucleic acid PCR clamp; BEAM: beads, emulsions, amplification and magnetics; DHPLC: denaturing high performance liquid chromatography; MALDI-TOF: matrix-assisted laser desorption ionization-time of flight; NGS: Next generation sequencing.

The term liquid biopsy was originally introduced to define circulating tumor cells (CTCs) but now includes circulating DNA and exosomes. Liquid biopsies are used for screening, to monitor treatment response, and to detect minimal residual disease after surgery. Liquid biopsies are considered important because genetic information from tumors can determine responses to certain treatments, such as epidermal growth factor receptor tyrosine kinase inhibitor (EGFR-TKI) in lung cancer. Lung cancer is a leading cause of cancer-related mortality, and 158,080 lung cancer deaths are estimated for 2016 in the United States alone [1]. In recent years, advanced understanding of molecular abnormalities in lung cancer has helped define disease subsets and led to development of specific molecular targets in the presence of driver mutations, thus providing invaluable information for cancer treatment. Most research and clinical trials in the past decade focused on EGFR mutations and on the abnormal fusion of the echinoderm microtubule-associated protein-like 4 (EML4) protein with anaplastic lymphoma kinase (ALK), which have been inhibited successfully with EGFR-TKI and crizotinib, respectively. These targeted therapies have become key components in lung cancer treatment and have demonstrated superiority to chemotherapy in terms of overall response rate (ORR), progression-free survival (PFS), and quality of life in patients with untreated non-small cell lung cancers (NSCLC) with sensitizing EGFR mutations [2–10]. However, these targeted therapies are based on mutation analysis *via* invasive examinations, including biopsy or cytology specimens obtained from bronchoscopy, computed tomography (CT)-guided biopsy, surgical resection, or drainage from malignant pleural effusions. Compared with liquid biopsies, tissue biopsies have drawbacks that limit the detection of targeted mutations. First, in advanced or metastatic NSCLC, not all cases have accessible tissues and are therefore unavailable for tissue biopsy [11]. Moreover, a failure rate of 5% to 10% is observed when commercially available tumor genotyping techniques are used, despite sufficient tissue availability [12]. Second, biopsies may not fully reflect tumor heterogeneity. A recent study used direct sequencing to identify EGFR mutations in 180 pairs of lung adenocarcinoma samples before treatment and demonstrated that the discordance rates in metachronous (primary tumors with matched distant metastases) and synchronous (primary lesions detected at different times) settings were 14.3% and 9.1%, respectively. Additionally, the discordance rate in patients with multiple pulmonary nodules was significantly higher (24.4%) [13]. Liquid biopsies do not have these limitations because less invasive techniques are used, and liquid biopsies are capable of capturing tumor heterogeneity and dynamically monitoring tumor molecular changes. The different platforms used in detecting EGFR mutations are illustrated at Figure 1.

## CIRCULATING TUMOR DNA

Circulating tumor DNA (ctDNA) is a subset of cell free DNA (cfDNA). The presence of small amounts of cfDNA in human plasma or serum was first discovered in 1948. Cell free ctDNA was then identified in the blood of cancer patients in 1977, and was successfully genotyped as a tumor marker after 17 years [14]. The mechanisms of ctDNA origin include apoptosis, cancer cell necrosis, and extracellular vesicles (EVs) secreted from cancer cells. Unlike genomic DNA, ctDNA in plasma is highly fragmented and ranges from 180 bp to 1000 bp in size when it originates from apoptosis and approximately 10,000 bp in tumor necrosis [10]. EVs are important in intercellular communication and contain large fragments ( > 10 kb) of double-stranded DNA with mutated *KRAS*, *p53*, or *EGFR* gene sequences [15, 16]. However, ctDNA represents a small fraction ( < 1.0%) of total cfDNA, and conventional sequencing approaches such as Sanger sequencing or pyrosequencing are not sensitive enough to detect EGFR mutations in cfDNA. Nevertheless, PCR-based diagnostics remain the key technique for detection and genotyping.

Many strategies have been developed to enhance the sensitivity of assays for detecting EGFR mutations. The amplification-refractory mutation system (ARMS)/Scorpion assay is widely applied as a ctDNA-based assay to detect various EGFR mutations [17–20]. ARMS primers use specific probes with increased allelic specificity and are efficient in differentiating wild-type and mutant DNAs with a low level of background if the 3′ base is mismatched. Therefore, specific mutated sequences are selectively amplified with complete efficiency when the primer is fully matched.

In contrast, peptide nucleic acids (PNAs) have been applied for suppressing/blocking the wild-type PCR product. Both PNA-mediated PCR and peptide nucleic acid-locked nucleic acid (PNA-LNA) PCR clamps were designed to enhance PCR sensitivity. PNAs and LNAs are high-affinity DNA analogues that hybridize with complementary DNA, and PNA-DNA hybrids are more stable than cDNA-DNA hybrids [21]. Because PNA oligomers cannot function as primers during PCR reactions, they prevent amplification of wild-type DNA templates and improve both the sensitivity and the specificity in discriminating single base-pair mismatches. LNAs also have higher affinity to DNA and are designed to recognize mutant sequences. In conjunction with PNA clamp primers, EGFR mutations can be detected in the presence of 100-fold to 1,000-fold wild-type EGFR backgrounds [22].

An alternative strategy is to divide each sample into 20,000 or more discrete subunits before PCR amplification. For example, digital PCR and beads, emulsions, amplification, and magnetics (BEAM) can detect mutations in cfDNA at frequencies as low as 0.01% [23]. Digital PCR allows partitioning of input DNA into droplets and independent PCR in individual droplets [24]. After amplification, each droplet is subjected to fluorescence measurement to decrease the ratio of ctDNA/germinal DNA, resulting in increased sensitivity. BEAM technology combines emulsion digital PCR and flow cytometry [25]. Emulsion PCR amplification is incorporated with magnetic beads and flow cytometry for highly sensitive detection of known genetic mutations, even at very low copy numbers. Because DNA is covalently bound to magnetic microbeads *via* streptavidin-biotin interaction, the PCR products generated in each droplet are affixed to microbeads at the end of the reaction, allowing subsequent separation and measurement of mutant variations by flow cytometry.

Both denaturing HPLC (DHPLC) and matrix-assisted laser desorption ionization-time of flight (MALDI-TOF) were developed for analyzing DNA mutations under the umbrella of mass spectrometry (MS). For detecting EGFR mutations by DHPLC, PCR products are subjected to ion-pair-reversed-phase liquid chromatography. Compared with homoduplex sequences without EGFR mutation, partially denatured heteroduplex PCR products with EGFR mutant DNA move through the chromatography column at different rates [26, 27]. MALDI-TOF MS distinguishes EGFR mutation by the different masses of primer extension products. It is characterized by a combination of amplification, primer extension reaction, and transfer of the reaction product to a chip containing specific matrix, thus allowing ctDNA detection with single-base specificity and sensitivity for a single DNA molecule [28, 29].

In contrast to techniques that detect known mutations, next-generation sequencing (NGS) can detect a subset of key genes and screen the entire tumor genetic code, with the potential to identify idiopathic resistant mechanisms. As with Sanger sequencing, NGS utilizes DNA polymerase to catalyze the incorporation of fluorescence-labeled nucleotides into a DNA template during sequential cycles of DNA synthesis. Instead of sequencing a single DNA fragment as in Sanger sequencing, NGS extends this process across millions of fragments in a massively parallel fashion [30]. Although the use of NGS has been reported for ctDNA-based EGFR mutation analysis [31, 32], its sensitivity of 2% allele frequencies for mutation detection in circulating DNA [33] is not as good as that of digital PCR. With the improvement of NGS platforms, including non-overlapping integrated reads (NIOR) [34], adding barcode sequences by adaptor ligation, and a digital sequencing platform that enables single molecular sensitivity [35], the false positives decreased and sensitivity increased. Recently, cancer personalized profiling by deep sequencing (CAPP-Seq) combined optimized library preparation for low DNA input masses by use of a panel of biotinylated DNA oligonucleotides designed through bioinformatics analysis targeting the mutated regions of interest. CAPP-Seq is an ultra-sensitive technology designed to monitor tumor DNA. This method can detect one molecule of mutant DNA from 10,000 molecules of healthy DNA. Using this platform, they successfully detected ctDNA in 100% of stage II-IV NSCLC patients and in 50% of stage I patients, with 96% specificity for the mutant allele down to ~0.02% [36]. To further improve the CAPP-Seq performance, an integrated digital error suppression (iDES) was developed that combines *in silico* elimination of background artifacts with molecular barcoding for efficient cfDNA recovery. EGFR kinase domain mutations can be detected by this method with 92% sensitivity and > 99.99% specificity at the variant level, and with 90% sensitivity and 96% specificity at the patient level [37]. However, NGS includes many complex processes, including template preparation, sequencing and imaging, and data analysis that determine its quality and its expense also limits its application in routine practice [38].

Other biological fluids, such as saliva and urine, are also used to detect EGFR mutations. Like blood, saliva contains a variety of biomolecules, such as DNA, mRNA, miRNA, proteins, and metabolites, that can be used as markers in predicting multiple cancers and systemic diseases [39]. Recently, we explored the clinical utility of saliva to detect EGFR mutations in NSCLC patients by developing an electric field-induced release and measurement (EFIRM) [40] composed of a polymer-based electrochemical chip with an array of 16 bare gold electrode chips as sensors. Paired probes, including detector probes and capture probes, are designed specifically for L858R and Exon 19Del. Detector probes are labeled with fluorescein isothiocyanate, and capture probes are copolymerized with pyrrole onto the bare gold electrodes by applying a cyclic square-wave electric field. After polymerization, the samples mixed with detector probes are transferred onto electrodes for hybridization. After anti-fluorescein antibodies conjugated to horseradish peroxidase are added, interactions between the 3,3′,5,5′-tetramethylbenzidine substrate and horseradish peroxidase occur and the amperometric signal is measured. The total detection time is less than 10 minutes and requires only 20 μL to 40 μL of the plasma or saliva sample. In a blinded test on saliva samples from 40 late-stage NSCLC patients, the receiver operating characteristic analysis indicated that EFIRM detected exon 19 deletions with an area under the curve (AUC) of 0.94 and detected the L858R mutation with an AUC of 0.96. The platform was also validated in another pilot study from China [41].

Like saliva collection, urine collection is a noninvasive procedure requiring no special facility or equipment apart from sterile collection containers. Two distinct DNA sizes can be identified from urine, with high-molecular-weight (HMW) DNA from urinary tract cells and low-molecular-weight (LMW) DNA was from circulation [42]. Recently, these short-length, tumor-derived DNA fragments were demonstrated to be filtered through the renal barrier and excreted into urine, and they can be used to detect *KRAS* in colon cancer [43]. Using a mutation enrichment PCR coupled with NGS detection, Reckamp et al [44] demonstrated that the sensitivity of the urine platform was 93% for T790M, 80% for L858R, and 83% for exon 19 deletions under a recommended volume of 90 mL to 100 mL of urine when the tissue result was used as a reference. At the American Society of Clinical Oncology (ASCO) Annual Meeting 2016, Wakelee et al [45] assessed EGFR status in tissue with a Therascreen EGFR test, in plasma by BEAM, and in urine by a quantitative short-footprint assay method using NGS. When tissue was used as the reference for EGFR genotyping, the positive percent agreement for T790M status between matched plasma and tissue was 81.5% (*n* = 195) and that between matched urine and tissue was 83.8% (*n* = 136). The study showed that ctDNA EGFR mutation testing from urine is a novel way to non-invasively detect the T790M mutations missed in biopsies because of tumor heterogeneity or inadequate sample quality. The sensitivity of these platforms is summarized in Table 1.

**Table 1:** Method or platform of CtDNA for detecting EGFR mutations and their associated sensitivity and application in lung cancer

**Platform**	**Sensitivity (% mutant DNA)**	**Targeting mutation**	**Reference**
Mass spectrometry	1%–10%	Known only	Arcila et al [110], Brevet et al [111].
NGS	1%–10%		Uchida et al [112]
Cobas, Therascreen, ARMS	1%–3%	Known only	Mok et al [80], Pasquale et al [113], Goto et al [17] Douillard et al [75] Li et al [114] Wang et al [19] Kuang et al [115]
PNA–LNA PCR clamp	2%	Known only	Kim [116] Kim [117].
TAM-Seq	2%	Known and new	Forshew [33]
EFIRM	1%–2%	Known only	Wei et al [40]
Digital PCR	<0.1%	Known only	Isobe et al [118]
BEAM	<0.1%		Taniguchi et al [119]
CAPP-Seq	~0.02%	Known and new	Newman et al [36]
iDES-enhanced CAPP-Seq	0.0025%	Known and new	Newman et al [37]

Abbreviations: TAM-Seq: Tagged-amplicon deep sequencing

CAPP-Seq: Cancer personalized profiling by deep sequencing

iDES: Integrated digital error suppression-enhanced CAPP-Seq

## CIRCULATING TUMOR CELLS

Numerous methods of enriching CTCs have been published and can be separated into label-dependent and label-independent strategies. The label-dependent method targets specific antigens on target cells by use of complementary molecules such as antibodies. The complementary molecules can be conjugated with magnetic beads or specific surfaces in a microfluidics platform. Among label-dependent techniques, immunomagnetic-based assays targeting EpCAM are most commonly used. Among the numerous EpCAM-based CTC detection technologies, the semi-automated CellSearch^®^ system is the most frequently used system and is the only one currently cleared by the U.S. Food and Drug Administration [46]. In addition to positively selecting target-cell populations, methods for negatively depleting off-target cells were also developed. Negative depletion against CD45-positive leukocytes is a preferred approach to isolate CTCs that lack adequate EpCAM protein expression. Enrichment selection methods utilizing anti-EpCAM antibodies have evolved through the introduction of microfluidic chips, such as CTC-chip, CTC-iChip^®^, and the silicon-nanopillar chip, to facilitate operational reliability and isolation efficiency. In CTC-chips, whole blood flows through a chamber embedded with 80,000 microposts coated with specific antibodies, which greatly increases the capture area [47]. The CTC-iChip employs continuous deterministic lateral displacement to remove nucleated cells from whole blood by size-based deflection utilizing a specially designed array. This inertial focusing, which lines the cells up and prepares them for precise magnetic separation and magnetophoresis, leading to sensitive separation in the immunomagnetic CTC isolation [48]. The silicon-nanopillar chip integrates ligand-receptor recognition, nanostructure amplification, and thermal responsive polymers, thus enabling highly efficient and selective cancer cell capture [49]

Label-independent enrichment methods separate CTCs based on physical rather than biological characteristics such as size, density, electrical properties, and inertial effect of flow. Filtering techniques utilize size differences and deformability characteristics of CTCs in comparison to blood cells, and the 3D parylene microfilter containing homogeneous pores enables direct CTC visualization/analysis on the filter [50]. The cell size in small-cell lung carcinoma (SCLC) and large-cell lung carcinoma (LCLC) ranges from an average of 7.2 μm to 15 μm in diameter as measured from biopsies [51] but CTCs from clinical samples can be smaller. The most frequently applied filtration methods are performed by use of Screencell^®^Cyto and ISET^®^ devices with track-etched polycarbonate filter pores measuring 7.5 μm to 8 μm in diameter and retain 85% to 100% of the tumor cells with only 0.1% of normal blood cells. Size-based filtration is convenient and is also applied to isolate lung cancer CTCs [52, 53], but its efficiency is limited because of false positivity arising from many leukocytes remaining on the membrane and smaller CTCs passing through it. This limitation is caused by the large range of CTC sizes in the same patient [54]. Traditional density-based gradient centrifugation methods using Percoll and Ficoll-Hypaque solutions separate the CTCs into the mononuclear cell fraction of blood, away from denser cells such as erythrocytes and granulocytes [55]. Various studies report that despite their popularity as inexpensive and reliable techniques, these methods have low CTC separation efficiency because the CTCs are still mixed with lymphocytes and monocytes. In addition, considerable numbers of tumor cells accumulate in the lower fraction instead of in the expected upper fraction after density gradient separation [54]. Thus, traditional density-based gradient centrifugation methods are not sufficient for precise CTC isolation. However, the robust operational benefit for primary CTC enrichment makes traditional density-based gradient centrifugation methods a good choice for combination with other label-free isolation methods, such as the OncoQuick^®^ system and the ApoStream^™^ system [56]. By combining a porous barrier that allows erythrocytes and some leukocytes to pass while retaining CTCs with density-based centrifugation, the OncoQuick^®^ system shows more effective enrichment [57]. However, elimination of contaminant monocytes is limited and the blood sample tends to mix with the gradient media if centrifugation is not performed immediately after applying the sample to the gradient media, thus restricting the usefulness of the samples if further enrichment techniques are not applied. Mammalian cells have a dielectric surface because they contain a variety of polarizable molecules, including proteins, peptides, and nucleic acids, and these dielectric properties are frequency dependent. Dielectrophoresis (DEP) applies a nonuniform electromagnetic field to the cell, and the cell responds to the DEP force by moving toward (positive DEP) or away from (negative DEP) the strong electromagnetic field [58]. Although low throughput limits its clinical utility as a device for isolating rare cells from blood, development of ApoStream^™^, which combines density gradient centrifugation and DEP, offers a continuous flow dielectrophoretic device for high throughput isolation and recovery of viable cancer cells from blood. Tran et al [56] used ApoStream^™^ to isolate CTCs from lung cancer and identified 12 EGFR mutations from 35 patients. Isolation and recovery of cancer cells from blood by use of ApoStream^™^ have been incorporated into numerous ongoing clinical trials. To improve throughput, a sustained 3D lateral DEP system was developed by Cheng et al [59]. Throughput can be effectively increased by proportionally increasing channel length (maximum flow rate: ~2.4 mL h^−1^, linear velocity: ~4 mm s^−1^). Spiral microfluidics focuses the positions of larger CTCs apart from smaller blood cells based on a combination of inertial and Dean drag forces in a spiral microfluidic device, enabling rapid and continuous isolation of viable CTCs. At larger flow rates, particles focused closer to the wall and larger particles are stabilized nearer to the channel center because of the inertial flow effect. Moreover, addition of curvatures introduces a secondary cross-sectional flow field perpendicular to the primary flow direction (Dean flow). Thus, at the spiral channel outlet region, larger particles such as tumor cells are focused and aligned near the inner wall, whereas smaller particles such as neutrophils and red blood cells occupy a lateral position near the outer wall [60]. The rapid processing time and the ability to collect CTCs from a large patient blood volume allows this technique to be used in a broad range of potential applications. Khoo et al [61] used the spiral microchip to isolate CTCs for detecting EGFR mutation and ALK translocation. A combination of label-dependent and label-independent techniques is also practicable. Yamamoto et al [62] describe the use of a magnetic capture column for rapid and efficient capture at a high flow rate combined with addition into the filter at a low flow rate. The combination was shown to decrease the time required for cancer cell capture, and the recovery rate increased from 64.7% when the filter was used alone to 80.7% in the combination method. The current strategies for CTC enrichment are summarized in Figure 1.

The main advantage of using CTCs as markers is that they are potentially the floating phase of solid tumors, providing good samples for immunohistochemically staining and *ex vivo* studies. In addition to EGFR mutation-targeted therapy, multiple examples of genotype-directed therapy producing dramatic responses in molecular subtypes of lung cancer are emerging, including *ALK* rearrangements, *ROS1* rearrangements, *MET* amplification, *BRAF* mutations, *HER2* mutations, and *RET* rearrangements [63]. In contrast to the difficulty of detecting these mutations in ctDNA [64], *ALK* status of CTCs can be assessed by immunohistochemistry or fluorescence *in situ* hybridization after isolation and characterization [65]. The applications of CTCs also include *ex vivo* mechanistic studies of drug resistance by generating primary cultures from CTCs known as CTC-derived xenografts.

However, the applications and studies involving CTCs in lung cancer genotyping remain few compared with those that utilize CtDNA because of some inevitable limitations: the heterogeneous CTC number among different cancers and the dynamic changes in CTC characteristics at different stages of the disease. CTCs may be reliably detected in most patients with metastatic prostate, breast, and colorectal cancers by use of the FDA-approved CellSearch technology, but this technology is limited in lung cancer because only approximately 10% of patients with NSCLC show ≥5 CTC/7.5 mL for enumeration [66]. Moreover, aggressive tumor cells often undergo epithelial-mesenchymal transition (EMT), which results in loss of epithelial markers such as EpCAM. Even in the same patient, the expression of epithelial markers on CTCs can change dynamically, resulting in ineffective detection by an EpCAM antibody-based enrichment technique [67].

## CLINICAL APPLICATION OF DETECTING EGFR MUTATIONS BY USE OF LIQUID BIOPSIES

### Concordance with tissue biopsies

When liquid biopsy was first used to detect EGFR mutations in NSCLC patients, concordance with tissue biopsy was the key concern. Recently, four meta-analysis studies were conducted to investigate the diagnostic value of ctDNA compared with that of tumor tissues. Luo et al [68] identified 20 studies with a total of 2,012 patients and demonstrated that the pooled sensitivity and specificity of cfDNA for detecting EGFR mutation status was 0.674 and 0.935, respectively. The methods used in these studies included direct sequencing, ARMS, denaturing high-performance liquid chromatography (DHPLC), peptide nucleic acid mediated polymerase chain reaction clamping, high-resolution melting (HRM), and digital PCR. Sub-group analyses showed that DHPLC and HRM had higher sensitivity than that of ARMS. Qiu et al [69] identified 27 eligible studies with a total of 3,110 participants and performed a meta-analysis. The pooled sensitivity and specificity were 0.620 and 0.959 (95% CI, 0.929 to 0.977), respectively. Overall analysis showed that ctDNA had high diagnostic accuracy and that ARMS was most useful for clinical practice. Mao et al [70] also demonstrated a similar result with a pooled overall sensitivity, specificity, and concordance rate of 0.61, 0.90, and 0.79, respectively, in a meta-analysis that included 25 studies with 2,605 patients. Another study by Qian et al [71] showed similar sensitivity (0.60) and specificity (0.94), where the sensitivities of PNA-LNA PCR, AS-PCR, and HRM were higher than those of ARMS and ME-PCR, but the specificity of ARMS was the highest among the other tests. Two multicenter diagnostic studies (Europe and Japan in ASSESS, and Asia-Pacific and Russia in IGNITE) also evaluated the utility of ctDNA for EGFR mutation testing in a real-world setting. In the ASSESS trial, both tissue and plasma samples were available from 1,162 patients with a sensitivity of 0.46 and a specificity of 0.974, but the results were improved in the Therascreen^®^ subgroup [72]. In the IGNITE trial, both tissue and plasma available from 2,581 patients showed similar sensitivity of 0.49.6 and specificity of 0.972, but included less data from Russian patients for unclarified reasons [73]. In summary, the specificity of different platforms is high (above 0.9), but the sensitivity is relatively low and varies from 0.4 to 0.7. The diagnostic value and platforms used in these studies were summarized in Table 2.

**Table 2:** Multicenter diagnostic trials and meta-analysis of circulating free DNA diagnostic value for detecting EGFR mutation status in NSCLC

**Trial (author)**	**Sensitivity**	**Specificity**	**Platforms**
Reck et al [72] 1162 patients (ASSESS trial)	0.46 (0.388–0.534)	0.974 (0.962–0.983)	ARMS, PNA-LNA
Han et al [73] 2,581 patients (IGNITE trial)	0.48 in Asia (0.458–0.534) 0.30 in Russia (0. 218–0.398)	0.972 in Asia (0.960–0.981) 0.932 in Russia (0.915–0.951)	Not mentioned
Jie et al [68] 20 studies 2012 cases	0.691 (0.569–0.790)	0.922 (0.878–0.951)	7ARMS, 3DHPLC, 2HRM, 2MEPCR, 1 AS-APEX, 1 digital PCR, 1ME sequencing,1 MEL, 1 PNA, 1 PNA-LNA
Qie et al [69] 27 studies 3110 cases	0.620 (0.513–0.716)	0.959 (0.929–0.977)	9ARMS, 3MEPCR, 2DHPLC, 2HRM, 2 AS-APEX, 1 digital PCR, 1BEAMing,1 Cobas, 1Sequenom,1 MEL, 1 PNA, 1 PNA-LNA, 1 Inhibiting PCR-quenching, 1Mutant-enriched sequencing
Mao et al [70] 25 studies 2605 case	0.61 (0.50–0.71)	0.90 (0.85–0.94)	6ARMS, 5direct sequencing, 4MEPCR, 3DHPLC, 3 PNA-LNA, 1 digital PCR, 1ME sequencing, 1WIP-QP, 1pyrosequencing
Qian et al [71] 27studies 3938cases	0.60 (0.57–0.62)	0.94 (0.93–0.95),	9ARMS, 3MEPCR, 2DHPLC, 2HRM, 2 PNA, 2 PNA-LNA, 2 AS-PCR, 1 digital PCR, 1ME sequencing,1 MEL, 1 NGS, 1 Deep sequencing

Abbreviations: HRM: High resolution melting analysis

ME-PCR: Mutant-enriched polymerase chain reaction

AS-APEX: Allele-specific arrayed primer extension reaction

MEL: Mutant-enriched liquid chip

WIP-QP: Wild inhibiting polymerase chain reaction (PCR) and quenched probe system

## PREDICTING PROGNOSIS AND DETECTING TKI-RESISTANT EGFR MUTATION

Because circulating DNA showed high concordance with tissue biopsies, some large clinical trials retrospectively investigated whether circulating EGFR-mutated DNA could be used to predict prognosis. In the Iressa Pan-Asia Study (IPASS) [8], EGFR mutations were assessed by use of tumor tissue-derived DNA (*n* = 91) and cfDNA from pretreatment serum samples (*n* = 194). A significant interaction occurred between cfDNA EGFR mutation status and treatment for PFS (*P* = 0.045). PFS was significantly longer and objective response rate (ORR) was borderline higher in patients receiving gefitinib treatment than in those receiving carboplatin/paclitaxel in the EGFR mutation-positive subgroup (PFS: hazard ratio [HR], 0.29; ORR: odds ratio [OR], 1.71; 75.0% *versus* 63.6%; *P* = 0.40). However, the high rate of false negatives (56.9%) resulted in a slight numerical advantage in PFS and ORR for gefitinib over carboplatin/paclitaxel in the EGFR mutation-negative subgroup [17]. The EURTAC trial demonstrated greater efficacy of erlotinib compared with chemotherapy for the first-line treatment of European patients with NSCLC harboring EGFR mutations in the tumor tissue [5]. EGFR mutations in ctDNA can be detected in 76 of 97 patients (78%), and the median OS was shorter in patients with the L858R mutation in ctDNA than in those with the exon 19 deletion (13.7 *vs*. 30.0 months; *P* < .001). Univariate analyses of patients with EGFR mutations in cfDNA identified the L858R mutation in tumor tissue or in cfDNA as a marker of shorter OS (hazard ratio, 2.70; *P* < .001) and PFS (HR, 2.04 [95% CI, 1.20 to 3.48]; *P* = .008) [74]. Another phase IV trial, NCT01203917, included plasma samples from 803 patients. The trial demonstrated that first-line gefitinib was effective and well tolerated in Caucasian patients with EGFR mutation-positive NSCLC. Median PFS was similar between mutation-positive tumors (9.7 m, 95% CI, 8.5 to 11.0) and mutation-positive plasma (10.2 m, 95% CI, 8.5 to 12.5) [75]. Another clinical trial was also designed to study whether plasma-based EGFR mutation analysis can predict tumor response. Bai et al [26] detected 81 EGFR mutations in 79 of 230 patient plasma samples (34.3%). Patients with EGFR mutations in plasma DNA had a PFS of 11.1 months (95% CI, 8.7 to 16.8) compared with 5.9 months (95% CI, 2.1 to 9.7) in patients with no EGFR mutations (*P* = 0.044). The dynamic changes in EGFR-mutated DNA detected by qualitative or quantitative analysis were also used to predict the treatment response to EGFR-TKI. Tseng et al [76] showed that failure to clear the plasma EGFR mutations after EGFR-TKI treatment for 10 weeks was an independent predictor of lower disease control rate, shorter PFS, and shorter OS. A similar result was reported by Lee et al [77], demonstrating that PFS was longer in patients with undetectable EGFR than in those with detectable EGFR mutations in blood after two months of treatment. The decrease rate in the semi-quantitative index of EGFR mutant DNA in plasma has demonstrated a correlation with percent tumor shrinkage [78], and Yang et al [79] further reported that high EGFR-mutated abundance in ctDNA showed better PFS than those with low EGFR-mutated abundance. Mok et al [80] further conducted a prospective analysis of blood-based EGFR mutation status assessment in the FASTACT-2 trial, which compared six cycles of gemcitabine/platinum plus sequential erlotinib or placebo. For patients with baseline EGFR mutations, median PFS was 13.1 months *versus* 6.0 months for erlotinib therapy and placebo. For patients with baseline EGFR mutations, median PFS was 7.2 months *versus* 12.0 months and median OS was 18.2 months and 31.9 months for positive EGFR mutation *versus* negative EGFR mutations by cycle 3, respectively (PFS: HR, 0.32; 95% CI, 0.21 to 0.48, OS: HR, 0.51; 95% CI, 0.31 to 0.84) [80].

Although NSCLC patients with EGFR mutations experience ORRs of 60% to 70%, almost all patients develop resistance to therapy with an average PFS of 9 months to 14 months. The mechanisms of acquired resistance to EGFR-TKIs include secondary mutations in EGFR, bypassed or alternative pathway activation, and histological/phenotypic transformation [81]. The most common cause of acquired resistance (60%) is the secondary mutation in EGFR in which methionine is substituted for threonine at position 790 (T790M) in exon 20. The third-generation EGFR inhibitors such as osimertinib (AZD9291), rociletinib (CO-1686), olmutinib (HM61713), EGF816, and ASP8273 are T790M mutant selective and EGFR wild-type sparing [82], leading to a need for noninvasive methods of T790M detection to guide the selection of therapy because of tumor heterogeneity and limited re-biopsies. Re-biopsy was not feasible in approximately 20% of cases because of difficult approaches, such as locations adjacent to central bronchi or vessels, miliary carcinomatosis [83], and the use of anticoagulants [84]. Moreover, both intratumoral and intertumoral heterogeneity undergo dynamic changes in relative populations of resistant clones over time as demonstrated in EGFR-mutant patients receiving more than one post-resistance biopsy and/or at autopsy [85, 86]. Many studies have demonstrated the applicability of liquid biopsies in detecting T790M and its correlation with treatment response. T790M was reported for the first time in 2005, but not until 2008 did Maheswaran et al [87] first detect the T790M mutation in circulating tumor cells from patients with EGFR mutations who had received tyrosine kinase inhibitors [87]. Wang et al [88] retrospectively investigated 135 patients with advanced NSCLC who showed PFS after EGFR-TKI for more than 6 months, for their status of EGFR-sensitive mutations and T790M mutation in matched pre-TKI and post-TKI plasma samples across multiple platforms. They demonstrated that D-PCR identified a higher frequency of T790M than ARMS did and that patients with pre-TKI T790M showed inferior PFS (8.9 months *vs*. 12.1 months, *P* = 0.007) and OS (19.3 months *vs*. 31.9 months, *P* = 0.001) compared with those without T790M. Similar results have been reported by Zheng et al [89], who investigated the correlation between plasma EGFR T790M ctDNA status and clinical outcome in advanced NSCLC patients with acquired EGFR-TKI resistance. Among patients receiving TKI treatment as the second line or later, the T790M ctDNA-positive group showed significantly shorter OS than that in the negative group. Because third-generation EGFR inhibitors such as osimertinib (AZD9291), rociletinib (CO-1686), and olmutinib (HM61713) show impressive efficacy, especially in T790M-positive patients with a response rate of approximately 50% and a median PFS ranging from 9.6 months to 13.1 months [90–92], a clinical trial targeting lung cancer harboring T790M as well as surveying the application of liquid biopsy for T790M was conducted. Although rociletinib usage has been stopped in the clinic because updated data revealed lower response rates than those initially reported and because of a negative vote from the FDA's Oncologic Drugs Advisory Committee, urine EGFR analyses can still be used to predict the treatment response in EGFR-TKI resistant NSCLC from the Tiger X trial [45]. Urine testing resulted in a sensitivity of 81.1%, as EGFR T790M could be detected in 142 of 175 patients identified by tissue analysis. When 22 inadequate tissue biopsies were included in the reference sample, urine testing identified almost as many T790M-positive patients. The similar investigator-assessed ORR (25% to 32% in 500 mg bid and 33 to 40% in 625 mg bid) and the median duration of response (mDOR) (9 months in 500 mg bid and 6.7 to 8.0 months in 625 mg bid) confirmed that urine testing can be used to predict treatment response to different doses of rociletinib as well as tissue samples [45]. Oxnard et al [93] performed both central tumor and plasma genotyping in 216 patients from 402 patients enrolled in the AURA Phase I escalation and expansion cohorts. When BEAM was used to analyze the plasma EGFR mutations, the sensitivity was 70% and the false-negative rate was 30% in plasma genotyping for T790M compared with tumor genotyping. PFS was also analyzed in patients classified by tumor and plasma genotyping. Tumor T790M positivity predicted a prolonged median PFS of 9.7 months, longer than that seen in tumor T790M-negative cases (*P* < 0.001). Although plasma T790M-positive status also predicts a prolonged PFS of 9.7 months, this survival is not significantly longer than that seen in plasma T790M-negative cases (8.2 months, *P* = 0.188). In plasma T790M-negative patients, tumor genotyping can distinguish patients with better and worse outcomes (tumor positive, 16.5 months *versus* tumor negative, 2.8 months, *P* < 0.0001). Among patients with plasma T790M positivity, those with tumor genotype showing positive T790M have a better outcome than those showing negative T790M in the tumor (9.3 months *versus* 4.3 months, *P* = 0.0002). This study suggests that patients can avoid a tumor biopsy for T790M genotyping if validated positive plasma T790M assays are available.

Although third-generation TKI agents were developed to have potency against tumors bearing EGFR-activating mutations in the presence of the T790M mutation, acquired resistance was developed with preliminary PFS estimates of ~10 months in T790M-mutated patients [91, 92]. By performing next-generation sequencing of cfDNA from seven patients who developed acquired resistance to AZD9291, Thress et al [94] identified the acquired EGFR C797S mutation in one patient and confirmed the role of C797S in mediating resistance to AZD9291 in a constructed mutant EGFR cell line. In two cases, tumor biopsies were available and the acquired C797S mutation was confirmed by targeted NGS because it was not detected in pretreatment tumors. Serial ddPCR profiling was performed on 15 subjects with advanced EGFR-mutant NSCLC before treatment and after acquired resistance to AZD9291. Pretreatment plasma ddPCR detected EGFR T790M mutations in 15 subjects and C797S mutations in none of the subjects. Upon developing AZD9291 resistance, six subjects acquired the C797S mutation, five subjects maintained the T790M mutation but did not acquire the C797S mutation, and four subjects lost the T790M mutation despite the presence of the underlying EGFR-activating mutation. This study demonstrated the application of NGS to exploring novel mechanisms of acquired resistance to third-generation EGFR-TKI by sequence analysis of cfDNA. The studies that applied ctDNA to predicting prognosis and to detecting TKI-resistant EGFR mutations in lung cancer are summarized in Table 3

**Table 3:** The application of CtDNA in predicting prognosis and detecting TKI-resistance-EGFR mutations in lung cancer patients harboring EGFR mutations

Author	Goal	Treatment	Case number	Platform	Conclusion
Goto et al [17]	Predicting prognosis by detecting EGFR sensitizing mutation	Gefitinib versus carboplatin/paclitaxel (IPASS trial)	194	ARMS	Significantly longer in PFS but borderline longer in ORR at EGFR^M^ (PFS: HR= 0.29; *P* < 0.001; ORR: OR= 1.71; 75.0% versus 63.6%; *P* = 0.40).
Karachaliou et al [74]	Predicting prognosis by detecting L858R and Exon 19 Del	Tarceva versus first-line chemotherapy	76	PNA clamp	Median OS was shorter in L858R group than exon 19 deletion (13.7 vs 30.0 months; P < .001) but not in the multivariate analysis
Douillard et al NCT01203917 [75]	Predicting prognosis by detecting EGFR sensitizing mutation	First- line gefitinib	803	ARMS	Similar Median PFS between mutation-positive tumor (9.7 m, 95% CI, 8.5–11.0) and plasma 1 (10.2 m,95% CI, 8.5–12.5)
Bai et al [26]	Predicting prognosis by detecting L858R and Exon 19 Del	Gefitinib after failure of Chemotherapy	102	DHPLC	Longer PFS in EGFR^M^ than EGFR^w^ (11.1 months, 95% CI, 8.7 to 16.8 vs. 5.9 month, 95% CI, 2.1 to 9.7)
Tseng et al [76]	Predicting prognosis by detecting L858R and Exon 19 Del	First-line use of TKI	72	PNA–ZNA PCR clamp	Shorter PFS (HR: 1.97, 95% CI: 1.33–2.91, *P* = 0.001) and OS (HR: 1.82 , 95% CI: 1.04–3.18; *P* = 0.036) in presence of EGFR^M^ after TKI treatment for 10 weeks compared with those without EGFR^M^
Yang et al [79]	Predicting prognosis by detecting EGFR sensitizing mutation and abundance	First-line or second-line use of TKI	73	DDPCR	1.Superior PFS (12.6 vs. 6.7 months, *P* < 0.001) and OS (35.6 vs. 23.8 months,) in EGFR^M^ 2. High EGFR^M^ abundance in ctDNA (> 5.15%) predicted better PFS (median, 15.4 vs. 11.1 months, *P* = 0.021).
Mok et al (FASTACT-2 study) [80]	Predicting prognosis by detecting EGFR sensitizing mutation	Six cycles of gemcitabine/platinum plus sequential erlotinib or placebo	238	RT-PCR	For EGFR^M^ patients with baseline; shorter PFS and OS in EGFR^M^ (+) cfDNA versus EGFR^M^ (−) cfDNA at cycle 3 patients (PFS, 7.2 versus 12.0 months, HR, 0.32; *P* < 0.0001); (OS, 18.2 vs. 31.9 months, HR, 0.51, *P* = 0.0066).
Wang et al [88]	Predicting prognosis by detecting EGFR T790M	First-line or second-line use of TKI	135	DDPCR ARMS	Pre-TKI treatment with T790M (+) showed inferior PFS (8.9 vs. 12.1 months, *P* = 0.007) and overall survival (OS, 19.3 vs. 31.9 months, *P* = 0.001) compared with those without T790M
Zheng et al [89]	Predicting prognosis by detecting EGFR T790M	TKI treatment at second-line or later	117	DDPCR	Patients with T790M (+) group had significantly shorter OS than the negative group (median OS: 26.9 months versus NA, *P* = 0.0489).
Wakelee et al [45]	Predicting prognosis by detecting EGFR T790M in urine	Rociletinib treatment in patients with EGFR^M^ NSCLC and acquired resistance to EGFR-TKIs (TIGER-X trial)	136	NGS	Objective response rate (ORR) and median duration of response (mDOR) are similar in T790M-positive urine (ORR, 32.0% in 500 mg bid and 40.7% in 625 mg bid; mDOR, 9 months in 500 mg bid and 8 months in 625 mg bid) and T790M-positive tumor (ORR, 25.0% in 500 mg bid and 39.4% in 625 mg bid; mDOR, 9 months in 500 mg bid and 7.9 months in 625 mg bid)
Oxnard et al [93]	Predicting prognosis by detecting EGFR T790M	Osimertinib (AZD9291) treatment in patients with EGFR^M^ NSCLC and acquired resistance to EGFR-TKIs (AURA Phase I)	216	NGS	ORR and median PFS were similar in patients with T790M-positive plasma (ORR, 63%; PFS, 9.7 months) or T790M-positive tumor (ORR, 62%; PFS, 9.7 months) results.

Abbreviations: PFS: Progression free survival. ORR: Overall response rate. HR: Hazard ratio

## LIQUID BIOPSY FOR LUNG CANCER GENOTYPING OTHER THAN EGFR MUTATION

Although ctDNA and CTCs have been broadly investigated for the correlation with tumor genotyping for EGFR mutation, their application in detecting other targets of genotype-directed therapy, including *ALK* gene rearrangements, *ROS1* gene rearrangements, *MET* gene amplification, *BRAF* gene mutations, *HER2* gene mutations, and *RET* gene rearrangements, remains limited. These mutations can be assessed by isolating CTCs and detection using fluorescence *in situ* hybridization or immunohistochemistry because specific antibodies and probes targeting these molecules are commercially available [65, 95–102]. Recently, EML4-ALK fusion [36, 98] and unreported fusions involving *ROS1* [36] were identified from plasma DNA by use of NGS platforms such as personalized cancer profiling by deep sequencing (CAPP-Seq). CAPP-Seq ctDNA analysis was also employed to investigate tumor heterogeneity and the associated mechanism of resistance to rociletinib. Chabon et al [103] identified one or more putative resistance mechanisms in 28 of 43 patients by serially collecting plasma before and after rociletinib therapy and demonstrated that *MET* copy-number gain was the most frequent mechanism in contrast to the most frequent C797S mutation that contributes to AZD9291 resistance [94]. Moreover, pre-existing copy-number gains in *MET*, *ERBB2*, and EGFR were significantly more common in patients with innate resistance. These studies underscore the potential of NGS to detect the uncommon drug-sensitive mutations and the alternative pathways contributing to EGFR-TKI resistance *via* ctDNA.

## GOLD STANDARD FOR DETECTING EGFR MUTATIONS AND ACQUIRED EGFR MUTATIONS AFTER RESISTANCE TO EGFR-TKI

In September 2014, the Committee for Medicinal Products for Human Use at the European Medicines Agency approved the use of ctDNA to assess the status of EGFR mutations when selecting EGFR-TKIs for patients in whom obtaining a tumor sample is not an option [104]. This update is applicable to all European Union member countries and will benefit patients who have locally advanced or metastatic NSCLC without available or evaluable tumor samples for EGFR mutation analysis. On June 1, 2016, the U. S. Food and Drug Administration approved the Cobas EGFR Mutation Test v2 (Roche Molecular Systems, Inc.), which uses plasma specimens as a companion diagnostic test for detecting exon 19 deletions or exon 21 (L858R) substitution mutations in EGFR to identify patients with metastatic NSCLC who are eligible for erlotinib therapy [105]. Based on a meta-analysis and other large clinical trials that demonstrate significantly longer PFS and higher ORR with EGFR-TKI than that in chemotherapy in the ctDNA EGFR mutation-positive subgroup [17, 70], the use of ctDNA to guide EGFR-TKI treatment is reasonable when tumor samples are not eligible for EGFR mutation assay.

**Figure 2:**
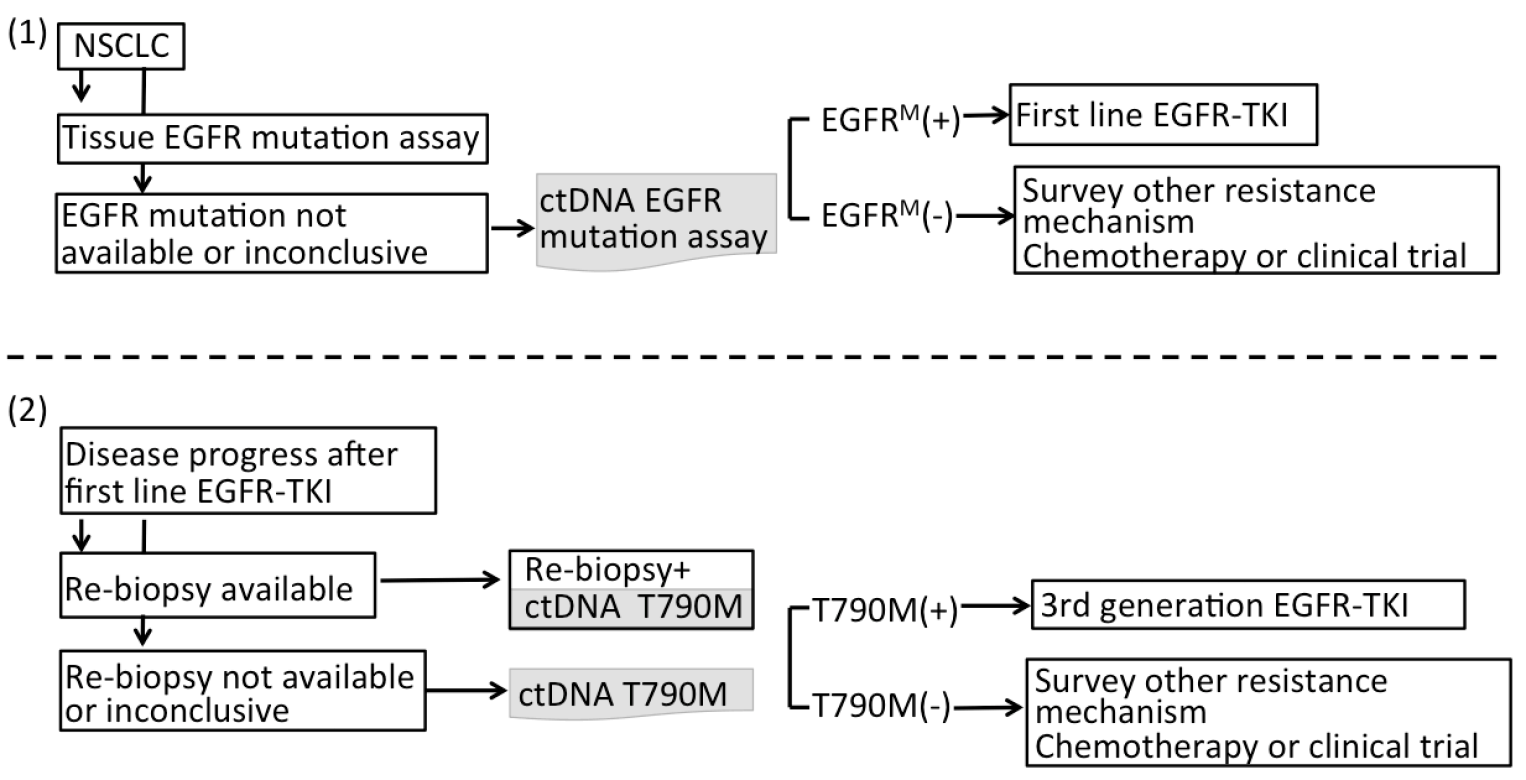
**A**. **The suggested paradigm for using plasma genotyping in clinical utility**. For NSCLC patients when tumor samples are not eligible for EGFR mutation assay or the result of EGFR genotyping is inclusive at initial diagnosis, ctDNA can be used to complement tissue biopsy guiding EGFR-TKI treatment. **B**. Among patients who developed resistance to first-line EGFR-TKI, liquid biopsies can compensate tumor biopsies because tumor biopsies themselves provide an incomplete window into the heterogeneous nature of acquired drug resistance.

Among patients who developed resistance to first line EGFR-TKI, many are too weak for re-biopsy, and a wide heterogeneity in resistance mechanisms may require its own therapeutic strategy [85, 106]. Therefore, liquid biopsies may compensate for the limitations of tissue biopsies. Liquid biopsies use less invasive techniques and are capable of capturing tumor heterogeneity. Although Oxnard et al [93] proposed a paradigm where plasma genotyping is used as a screening test for T790M, before performing an EGFR resistance biopsy, the high false-positive rate (30%) in plasma DNA and the outcomes of these patients depended on tumor genotyping. This dependence caused clinicians to become concerned about whether positive plasma DNA can be used to guide third-generation TKI use for patient developing drug resistance. In a recent update, the consensus statement on optimizing the management of EGFR mutation-positive NSCLC is that tissue-based molecular analysis remains the gold standard for establishing the initial diagnosis, as well as for evaluation of TKI resistance [107]. Recently, Sundaresan et al [108] compared the T790M genotype from tumor biopsies with an analysis of simultaneously collected CTCs and ctDNA. T790M genotypes were successfully obtained in 30 tumor biopsies (75%), 28 CTC samples (70%), and 32 ctDNA samples (80%). Although CTC-based and ctDNA-based genotyping failed to detect T790M in 20% to 30% of all cases, both assays together enabled genotyping in all patients with available blood samples and identified the T790M mutation in 14 patients (35%) in whom the concurrent tumor biopsy was negative or indeterminate. The finding was compatible with that of Oxnard et al [93, 109], wherein cases that were T790M negative in the tumor but were T790M positive in the plasma were further studied by use of orthogonal plasma genotyping assays such as ddPCR or Cobas^®^, and 78% of these cases could be confirmed as positive. Therefore, liquid biopsies can compensate for tumor biopsies because individual tumor biopsies alone provide an incomplete window into the heterogeneous nature of acquired drug resistance. Only their correlation with the clinical response to third-generation EGFR inhibitors may ultimately provide a true “gold standard” for T790M genotyping. The suggested paradigm for the use of plasma genotyping to complement tissue genotyping is summarized in Figure 2.

## CHALLENGES OF LIQUID BIOPSY GENOTYPING ON THE PATH TO CLINICAL UTILITY

Though remarkable progress in the use of liquid biopsy has been made in recent years, several challenges remain to be overcome for its application in clinical routine practice of lung cancer. Many studies have aimed to detect and/or characterize CTCs or ctDNA in lung cancer patients; the question is which of these two approaches will become a better platform for managing these patients in the era of precision medicine. It seems as though more scientific studies exist supporting the use of ctDNA for profiling and characterization of lung tumor molecular alterations as well as for monitoring therapies and identifying mutations associated with acquired drug resistance. The most important issue is that plasma samples can be collected and analyzed without requiring prior enrichment, and there is no need to isolate a rare cell population. Compared to other cancers such as prostate and breast cancer, the number of CTCs in lung cancer is relatively low [105]. However, one important limitation of ctDNA is that *in situ* and morphological analyses using FISH and ICC (including ALK or ROS1 status) cannot be performed with ctDNA. Though the combination of CTC and CtDNA has been applied in detecting T790M [108], the application of these two platforms together in lung cancer genotyping including EGFR, ALK, ROS, and MET amplification remained limited. The major problem is that there are many diverse techniques in each platform, and standardization is more complicated and requires more effort. Second, though the newly developed ctDNA platforms such as digital PCR, BEAMing, and advanced NGS platform showed higher sensitivity compared to conventional PCR-based platforms such as the Cobas EGFR kit and TheraScreen^®^ EGFR plasma PCR kit, which were approved by FDA, Europe and China respectively (Table 1), the specificity of these platforms was relatively low [93]. More effort should be expended on optimizing these diverse technologies of ctDNA analysis and standardizing different platforms, and appropriate analytical and clinical validity needs to be demonstrated to control the pre-analytic phase and obtain robust and reproducible results. Finally, no randomized controlled trial demonstrates that liquid biopsy can be used to guide treatment strategy or compliment tissue biopsy. Critical clinical standards need to be established, and well-designed and sufficiently powered multicenter clinical trials involving large cohorts of patients and controls are required to validate ctDNA as clinical tools. In addition, activation of alternative pathways such as epithelial mesenchymal transition or histologic transformation to small cell carcinoma, which also mediate resistance to EGFR TKI, cannot be detected by the currently available liquid biopsy platforms. Therefore, tissue biopsy cannot be replaced by liquid biopsy for diagnosis and treatment in advanced NSCLC but the combination of tissue and liquid biopsy will offer additional information that will help to treat lung cancer patients better in the view of tumor heterogeneity and predicting treatment response.

## CONCLUSIONS

The above studies regarding liquid biopsies underscore their potential utility in lung cancer genotyping. With the paradigm shift brought by liquid biopsies in lung cancer treatment, additional qualifications combining clinical pathology, molecular pathology, and molecular biology will be required in the field of precision medicine. Changes in practice in oncology caused by judicious use of liquid biopsy potentially improve patient outcomes by expediting treatment decisions, predicting treatment response, and anticipating tumor relapses that are not yet visible on imaging.
